# Role of LDL level over hospital stay length of surgically treated coronary artery and obstructive peripheral arterial disease patients

**DOI:** 10.1186/1749-8090-10-S1-A228

**Published:** 2015-12-16

**Authors:** Ersin Çelik, İsmail Yürekli, Ufuk Yetkin, Habib Çakir, Köksal Dönmez, Metin Gümüş, Rahika Durusoy, Ali Gürbüz

**Affiliations:** 1 Department of Cardiovascular Surgery, Katip Celebi University Izmir Ataturk Training and Research Hospital, Izmir, Turkey; 2 Department of Public Health, Medical Sciences Faculty, Ege University, Bornova, 35040 Bornova/İzmir, Turkey

## Background/Introduction

Atherosclerosis is the most common and most important risk factor for cardiovascular diseases.

## Aims/Objectives

Between dates January 2007 and December 2010, 868 coronary artery disease, and 268 peripheral vascular disease patients who were treated surgically at our clinic were investigated.

## Method

Mean age of 868 coronary artery disease patients were 63,86 ± 11,17 (between 21-91 years) and 268 peripheral arterial disease patients were 65,44 ± 10,37 (between 21-92 years).

## Results

Between 868 patients underwent surgery for coronary artery disease, LDL cholesterol level of 518 patients were >100 mg/dl and 350 patients were < 100 mg<7 mg/dl. Mean hospital stay length of patients was 6,8 days and 6,19 days, respectively. This difference was significant (p < 0.05).Between 268 patients underwent surgery for peripheral arterial disease, LDL cholesterol level of 177 patients were >100 mg/dl and 91 patients were < 100 mg<7 mg/dl. There was any significant correlation between LDL cholesterol levels and intensive care or hospital stay length (p > 0.05).

## Discussion/Conclusion

We believe that, precise examination of preoperative risk factors and providing adequate pre and per operative medication will significantly reduce surgical morbidity rates, intensive care unit, and hospital stay lengths of coronary and peripheral arterial disease patients.

## Consent

Written informed consent was obtained from the patient for publication of this Case report and any accompanying images. A copy of the written consent is available for review by the Editor-in-Chief of this journal.

